# The dual role of red deer in yellow rockrose seed dispersal and predation in Mediterranean Spain

**DOI:** 10.1111/plb.70102

**Published:** 2025-10-03

**Authors:** R. Fernández‐Fuerte, P. J. Garrote, E. Virgós, J. M. Fedriani

**Affiliations:** ^1^ Estación Biológica de Doñana, Consejo Superior de Investigaciones Científicas (CSIC) Sevilla Spain; ^2^ Departamento de Biología, Geología, Física y Química Inorgánica, Escet Universidad Rey Juan Carlos Madrid Spain; ^3^ Centro de Investigaciones Sobre Desertificación CIDE, CSIC‐UVEG‐GV, Carretera de Moncada a Náquera Moncada Spain

**Keywords:** *Cervus elaphus*, *Cistus halimifolius*, Doñana National Park, endozoochory, faecal analysis, mutualism–antagonism, seed germination

## Abstract

- Endozoochory, seed dispersal after animal ingestion, is a mutualistic plant–animal interaction that often involves substantial costs (e.g. ingested seed destruction). However, few studies have simultaneously comprehensively assessed the costs and benefits. We investigated the interaction between overabundant ungulates and the Mediterranean shrub *Cistus halimifolius* in Doñana National Park.
- We evaluated frequency of ungulate visits to fruiting *C. halimifolius* individuals using camera traps. We then assessed seed occurrence and germination success via faecal analysis and germination experiments.
- Camera traps revealed that the red deer, *Cervus elaphus*, were the principal consumer of *C. halimifolius* fruits (85.7% of 333 ungulate visits). Although red deer frequently visited *C. halimifolius*, only 4.5% of faecal samples (*n* = 246) contained seeds, likely because red deer consumed immature fruits whose seeds were then fully digested and thus undetectable. Indeed, all seeds recovered within deer faeces were fully developed, mature seeds. 17% of the mature deer‐ingested seeds (19 out of 114) germinated, a lower proportion than in control mature seeds (40%, *n* = 168).
- Mature seed passage through the deer digestive tract reduced *Cistus* seed germinability. Despite low germination rates, the highly mobile deer are potential long‐distance seed dispersers of *C. halimifolius*, promoting population gene flow and the (re)colonization of vacant habitats. The interaction between *C. halimifolius* and red deer involves substantial costs, which likely changes spatio‐temporally and also depend on densities of the interacting species. Future studies should quantify such changes and identify whether and how such pervasive interaction in Mediterranean ecosystems shifts along a mutualism–antagonism continuum.

## Graphical abstract

Red deer play a mostly antagonistic and predatory role on *Cistus halimifolius* seeds, but here, it has been demonstrated, for the first time, their overlooked mutualistic potential role as long distance disperser.

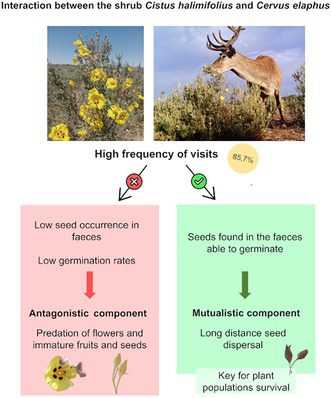

## INTRODUCTION

Species' interactions are often simplified into two categories: antagonistic or mutualistic. However, such interactions are frequently complex, with conflicting and overlapping interests, potentially resulting in both negative and positive net outcomes (Bronstein 1994, 2015; Fedriani & Delibes 2011). The net outcome of a particular interaction is highly variable and depends on biotic and abiotic factors (Perea *et al*. 2013; Bronstein 2015; Dracxler & Kissling 2022). Hence, it depends strongly on the ecological context and a mutualism–antagonism continuum (Perea *et al*. 2013; Chamberlain *et al*. 2014; Bronstein 2015). Despite the dynamic and heterogeneous nature of species interactions, a rigorous assessment of the associated costs and benefits, and thus ultimate net effects, is missing in most such interactions.

Seed dispersal is one of the most important ecological processes in plant population dynamics, shaping distribution of future individuals in locations with variable conditions for establishment (Tiffney 2004). Long‐distance seed dispersal (while rare) facilitates gene flow, the connection of subpopulations, distribution range expansion, and the (re)colonization of vacant habitats (Bacles & Ennos 2008; Nathan *et al*. 2008; González‐Varo *et al*. 2021). Seed dispersal via animal ingestion (i.e., endozoochory) represents a mutualistic relationship, where both plant and animal benefit: the plant gains mobility and, sometimes, germination, while the animal receives nutrients and water (Malo & Suarez 1995; Herrera 2002; Corlett 2021; Beckman & Sullivan 2023). Although passage through the animal's digestive tract often increases the speed and germination capacity of ingested seeds (Fedriani & Delibes 2009; Mancilla‐Leytón *et al*. 2011; Milotić & Hoffmann 2016), endozoochory also represents an important cost for the plant (e.g., Traveset *et al*. 2007; Fedriani & Delibes 2011; Perea *et al*. 2013). Seeds are impacted during ingestion and passage through the animal gut, and a fraction of them (mostly immature seeds) is destroyed (Traveset *et al*. 2007).

Investigations of endozoochorous systems have often focused on plants with fleshy fruits (Herrera 2002; Fedriani *et al*. 2012; Jordano 2014). However, dry fruits are often ingested and their seeds dispersed by fish, bird, rodent and ungulate species (Malo & Suarez 1995; Corlett 2021; Green *et al*. 2022; Navarro‐Ramos *et al*. 2024). Ungulates are important shapers of dry‐ and fleshy‐fruited plant communities (Augustine & McNaughton 1998) by acting as both antagonists (consuming leaves, flowers, buds, mature and immature seeds; Lecomte *et al*. 2016; Dracxler & Kissling 2022) and mutualists (dispersing mature seeds; Ramos *et al*. 2006; Pellerin *et al*. 2016). The net outcome of plant–ungulate interactions varies depending on the plant and ungulate species (Jaroszewicz *et al*. 2013; Perea *et al*. 2013; Garrote *et al*. 2023). Even for a given plant–ungulate pair, the sign and strength of interaction is often altered by factors such as the density of ungulates, plant sex, or plant ontogenetic stage (Lecomte *et al*. 2016; Muñoz‐Gallego *et al*. 2019, 2023). For instance, immature seeds are soft, and thus more susceptible to damage in the ungulate digestive tract, whereas mature seeds are more likely to be dispersed by ungulates, giving rise to a mutualistic association (Ishikawa 2011; Viero *et al*. 2018).

Here, we investigate the costs and benefits of the interactions between the Mediterranean shrub *Cistus halimifolius* L. and several ungulate species, including red deer (*Cervus elaphus*) and wild boar (*Sus scrofa*). Both *C. halimifolius* and ungulates are extremely abundant in the southern Iberian Peninsula (Díaz Barradas *et al*. 1999; Zunzunegui *et al*. 2002; Bugalho & Milne 2003; Carranza 2017). Surprisingly, however, very little is known concerning the strength and sign of this interaction. Previous studies found that other Cistaceae–ungulate interactions are pervasive and intense in Mediterranean environments (Ramos *et al*. 2006; Mancilla‐Leytón *et al*. 2015; Lecomte *et al*. 2016). Thus, we predict that the magnitude of the interaction between *C. halimifolius* and ungulates would also be strong. We expected that areas with overabundant ungulate populations might have a negative impact on development of this plant, via fruit and seed predation, and thus reduce plant growth and reproduction (Mancilla‐Leytón *et al*. 2011; Lecomte *et al*. 2016, 2017). Nevertheless, red deer could also be a potential seed disperser, as found for other Cistaceae species (Malo & Suarez 1998; Ramos *et al*. 2006). To address this, we selected two sites within a Mediterranean ecosystem of southwest Spain where *C. halimifolius* and mammalian ungulates are very abundant. We combined camera trapping, ungulate faecal analysis, and germination experiments to evaluate costs and benefits of the interaction between this shrub and its main ungulate consumer. Specifically, we addressed the following questions: (i) what is the dominant ungulate consuming *C. halimifolius* and does this vary between study sites; (ii) does seed consumption by ungulates affect seed percentage and speed of germination; and (iii) can this plant–ungulate interaction be characterized in terms of costs and benefits?

## MATERIAL AND METHODS

### Study area and sites

**Fig. 1:**
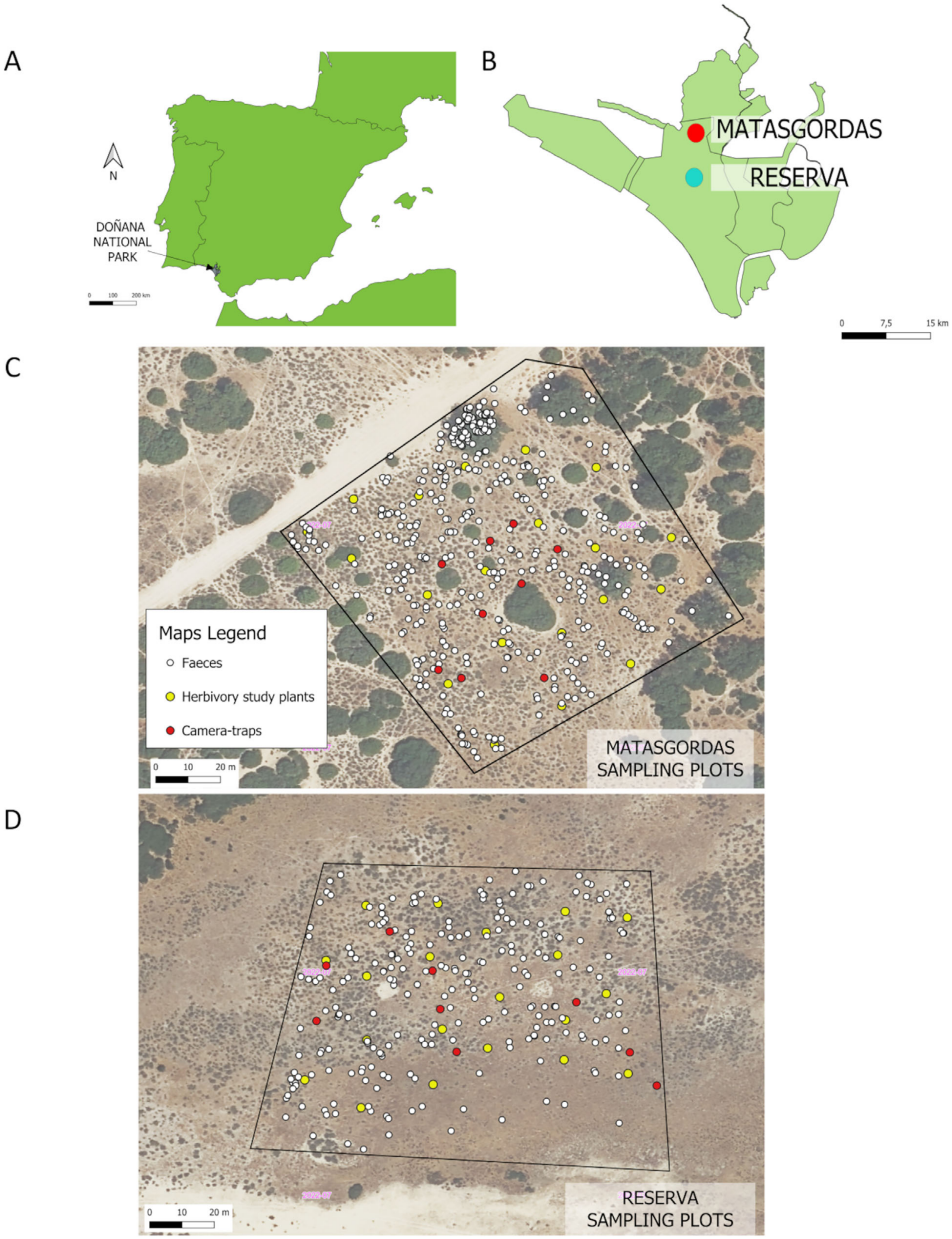
The study plots. (A) Location of the Doñana National Park within SW Spain. (B) Doñana National Park with the two study sites, Matasgordas (red) and Reserva (blue). Sampling points of faecal samples (white), selected plants (yellow) and camera traps (red) in Matasgordas (C) and Reserva (D).

The study was carried out between 2022 and 2024 in the Doñana National Park (SW Spain), on the west bank of the Guadalquivir River (Fig. 1A). The area is characterized by a sub‐humid Mediterranean climate (537.7 ± 30.7 mm annual precipitation) with dry (8.4 ± 1.4 mm) and hot summers (38.5 ± 0.3°C maximum, 10.7 ± 0.2°C minimum average temperature; Data from Natural Processes Monitoring Group; Garrote *et al*. 2024). Most precipitation occurs between October and March (80%), with little to no rainfall during the summer (Muñoz‐Reinoso 2023). Doñana is an anthropogenic and fragmented landscape that comprises three main ecosystems: Mediterranean scrubland, marshes, and mobile dunes (Garrote *et al*. 2022a).

We selected two similar‐sized (~1 ha) tetragonal plots, 10 km apart, of scrubland (Matasgordas and Reserva) with the dominant plant being *Cistus halimifolius* (Fig. 1B). According to the Index of Kilometric Abundance for 2005–2022 (Natural Processes Monitoring Group) ungulates such as red deer (*Cervus elaphu*s) and wild boar (*Sus scrofa*) are abundant in Doñana (Table 1). These surveys were conducted by car, over 14.8 and 14.4 km in Matasgordas and Reserva, respectively. They took place in the afternoon and evening in spring and autumn, to assess pre‐breeding and post‐breeding populations, respectively. Although fallow deer are present in both study sites, no individuals were detected in these surveys. Matasgordas, in the northern section of the National Park, has been highly affected by human activities (e.g. cultivation, livestock ranching, hunting) and herbivory pressure. The area has two clearly distinguished habitat types: scrubland and old‐field. Our study plot is a scrubland dominated by *Pistacia lentiscus* and variable densities of shrubs such as *C. halimifolius, Stauracanthus genistoides, Cistus* spp., *Chamaerops humilis, Daphne gnidium* and *Asparagus aphyllus. Quercus suber*, *Olea europaea* var. *sylvestris, Fraxinus angustifolia, Pinus pinea* and *Pyrus bourgaeana* trees are also relatively abundant. Livestock ranching in Matasgordas is currently very limited as it was removed when the area was expropriated in 1998. Three ungulate species (red deer, fallow deer *Dama dama*, and wild boar) are present in this study site, red deer and the wild boar being the most abundant (Table 1). Our study plot in Reserva was in the ecotone between the scrubland and the marshes, known locally as “Vera”. This ecotone is dominated by prairies, with sparse patches of *C. halimifolius*, *S. genistoides*, *C. humilis* and some *Q. suber*, *O. europaea* var. *sylvestris*, and *P. bourgaeana* trees (González‐García *et al*. 2022). Livestock ranching is locally intensive, mainly cattle and horses (Garrote *et al*. 2022a). The ungulate species present in this study site are horses, cattle, red deer, fallow deer, and wild boar (Table 1).

**Table 1:** Mean Index of Kilometric Abundance (IKA) of wild vertebrate herbivores in both study sites from 2005 to 2022.

herbivore species	matasgordas	reserva
Rabbit (*Oryctolagus cuniculus*)	13.75 ± 2.05 (*N* = 42)	0.22 ± 0.05 (*N* = 42)
Iberian hare (*Lepus granatensis*)	0.03 ± 0.01 (*N* = 18)	0.23 ± 0.06 (*N* = 18)
Red deer (*Cervus elaphus*)	8.52 ± 1.56 (*N* = 18)	3.71 ± 0.38 (*N* = 18)
Wild boar (*Sus scrofa*)	0.43 ± 0.08 (*N* = 42)	0.43 ± 0.08 (*N* = 42)
Cattle (*Bos taurus*)	‐^a^	68.0 ± 1.4 (*N* = 67)
Horse (*Equus ferus caballus*)	‐^a^	139.7 ± 1.8 (*N* = 61)

IKA is the average number (±1 SE) of individuals detected per survey (the number of surveys is shown in parenthesis). Surveys were conducted by the Natural Processes Monitoring Group (Doñana Biological Station). The length of surveys was 14.8 and 14.4 km in Matasgordas and Reserva, respectively. Cattle and horse are not present in Matasgordas, and in Reserva, the number of calves and foals is not considered (Data from Doñana Biological Station and Doñana Natural Space). Although exact IKA data are not available for this sampling, fallow deer is present at both study sites.

a Only anecdotal presence.

### Study species

The yellow rockrose, *Cistus halimifolius* L. (synonym *Halimium halimifolium* L. Willk; Cistaceae) is a woody shrub mainly found in the Western Mediterranean basin (Díaz Barradas *et al*. 1999). This species is an obligate seeder, and one of the main components of the scrub vegetation in the Doñana area, being dominant on sand ridges (Zunzunegui *et al*. 2000; Suárez‐Esteban *et al*. 2013; Arroyo‐Correa *et al*. 2021). Morphologically, *C. halimifolius* is a densely branched shrub up to 2 m high, with opposite, obovate or elliptical leaves, which are tomentose when young and greener with age. The flowers, yellow with five petals, are distributed in cymose inflorescences, and the calyx is also a pentamer (Díaz Barradas *et al*. 1999). The fruit is an ovoid dehiscent capsule (4–8 mm diameter), with stellate hairs and three valves, that contains 30 and 50 seeds of about 1 mm diameter. Mature seeds are polyhedral, with a rough, dark grey surface and are extremely hard (Navarro & Gálvez 2001). Immature seeds are more rounded, whitish green, with a smooth surface and much softer and less resistant to erosion and abrasion (pers. obs.). Plants flower from March to June, and fruits ripen between June and August (Navarro & Gálvez 2001). Flowering, fruiting and fruit ripening are asynchronous between and within populations, as well within individuals. Thus, the presence of inflorescences, immature fruits, and mature fruits are often simultaneously observed in the same individual (unpubl. data).

*Cistus halimifolius* seed is dispersed over short distances by barochory (Bastida & Talavera 2002; Rodriguez *et al*. 2017). Surprisingly, despite *C. halimifolius* pervasiveness and high density in many Mediterranean ecosystems, very little is known about its potential long‐distance dispersal mechanisms (but see Mancilla‐Leytón *et al*. 2015; García *et al*. 2023). Nevertheless, *C. halimifolius* structures have been found in stomach and faecal samples of ungulates (Venero 1984). Ungulates and Cistaceae have coexisted in the Western Mediterranean for at least 1.8–1.9 million years. Ungulates feed on most plant parts, including leaves, flowers, immature and ripe fruits. As a result, Cistaceae has evolved endozoochory (Ramos *et al*. 2006; Lecomte *et al*. 2016), with many small seeds and a rough and hard outer layer when mature to protect the endosperm. These seed traits likely reduce damage from mastication and increase the probability of mature seed survival (Thanos *et al*. 1992; Mancilla‐Leytón *et al*. 2011). In addition, seeds of *C. halimifolius*, like those of other members of this family, exhibit dormancy that is broken by exposure to heat, allowing them to germinate post‐fire. Passage through the digestive tract of ungulates also enhances seed germination (Lecomte *et al*. 2016); however, immature soft seeds are subject to damage when ingested by ungulates, often leading to seed mortality.

### Camera trapping

To estimate the intensity of the *C. halimifolius*–ungulate interaction during fruit development and ripening (mid‐July), camera traps (LTL ACORN 5310A, detection range 18 m) were placed within each study plot (Fig. 1C), with nine cameras in each plot, to maximize the area covered by the cameras (Fig. 1C,D). Cameras were attached to a wooden pole 1 m above the ground, and 2 to 3 m to the targeted *C. halimifolius*. Cameras were set to take a burst of three photos with a 0‐s time lapse when movement was detected, with a 1 min interval before the next burst. Each group of three photos was considered as one visit, which was the unit used to quantify intensity of the interaction. A visit may include any animal activity around (i.e., within 1 m) the plant, either browsing or merely approaching it, with such activity type being recorded. If there was more than one individual of the same species in the three photo burst, the highest count of individuals was considered. Although the prolonged presence of a single individual in a plot might be recorded as multiple distinct visits, this approach provides a better proxy of the interaction intensity than ignoring successive visits by the same individuals (e.g., Selwyn *et al*. 2020). The cameras were active for 10 days. The software CPW Photo Database (v. 4.3.0.6) was used to examine the photographs and identify all vertebrates, whether or not they used the focal *C. halimifolius* individual, together with the date and time of day. Photos were interpreted in relation to the animal's proximity to the plant and its intentions (e.g., if it had its head down or was just passing by), referring to plant use as consumption of any part of the plant. The intensity of the interaction between ungulates and *C. halimifolius* was estimated as number of ungulate visits per number of camera traps per day.

### Collection of ungulate faecal samples

To quantify the frequency of *C. halimifolius* seeds in ungulate faeces, we systematically conducted faecal surveys in the two study plots (Fig. 1C,D). Ungulate faeces often comprise dispersed seeds (e.g., Perea *et al*. 2013). Red deer faeces are a grouping of (20–30) oval pellets, each 1.0 to 2.5 cm long and 0.8 to 1.4 cm wide, brownish black and hard. These small pellets are usually fused together, resulting in pellet groups. Although some red deer pellets may be isolated, we focussed our sampling exclusively on pellet groups, defined as ≥30 pellets within a circular plot of 50 cm diameter (Suárez‐Esteban *et al*. 2013). Red deer and fallow deer (*Dama dama*) faeces are very similar or even indistinguishable, so in this study it was assumed that they were red deer, due to their abundance in our study sites (Braza & Alvarez 1987; Muñoz‐Reinoso 2017). Wild boar are also abundant, feeding on animal and plant (fruits, seeds, roots, etc.) items. Their faeces are units of 5 cm in diameter which form larger masses, from brown to black and with varied composition. Other abundant ungulates in Doñana are domestic livestock such as horses (*Equus ferus caballus*) and cattle (*Bos taurus* L.) (Table 1). Horse faeces are oval, at least 4 cm thick, smaller than a tennis ball, greenish brown and oblong with rounded ends, singly or in amorphous units. Cattle faeces are bigger, of pasty consistence, flattened in circular piles up to 50–80 cm in diameter (McGranaghan *et al*. 1999; Purroy & Varela 2003; Richarz 2007).

Faeces collection was conducted during summer 2022. The first transect was in mid‐June 2022 and repeated on a weekly basis until the end of July, coinciding with the end of *C. halimifolius* fruiting, with six transects in total (Tables S1 and S2). Each transect had one starting point per plot, and followed a non‐fixed zigzag path to the opposite end point of the plot (Fig. S1; Garrote *et al*. 2022b). This was repeated weekly, and each week the starting point was changed, by rotating them clockwise, as did the orientation. Consequently, transects were perpendicular to those of the previous week and, thus, the plot was homogeneously covered (e.g. Garrote *et al*. 2022b). In the first week the plot was swept, collecting fresh or recent faeces and removing old ones, in order to obtain a clean plot for the following week.

During the transects, all fresh or relatively fresh mammal ungulate faeces within a radio of ~5 m from the observer's trajectory (i.e., width ~10 m) were collected and stored in paper envelopes (10 × 14.5 cm) in a dark, dry place in the laboratory, in order to minimize the effect of storage on sample preservation. The date, coordinates, and ungulate species were recorded (see Table S2). On average, 70 ± 3.9 and 38.3 ± 4.5 faeces were found during each sampling in Matasgordas and Reserva, respectively. Overall, we collected 641 ungulate faeces. Specifically, 406 and 7 samples of deer and wild boar in Matasgordas; and 185, 35 and 8 samples of deer, horse and cattle in Reserva.

### Faeces analyses

Overall, we analysed 288 ungulates faecal samples (264, 12, 6 and 6 samples of red deer, horse, cattle and wild boar, respectively). Faeces were analysed in two separate periods, one in 2023 and one in 2024. Only a subset of the total samples was analysed in order to optimize analysis time, while still representing a significant portion of the overall sample set. In 2023, a random subsample of 130 samples (21% of total) of different ungulate species was analysed (106, 12, 6 and 6 samples of deer, horse, cattle and wild boar, respectively). In 2024, an additional different random subsample of 158 faecal samples of deer (25% of total) was analysed to find the most *C. halimifolius* seeds for the germination experiment, given that red deer are the major consumers of the plant (287 red deer visits out of 333 ungulate visits in camera trapping analyses).

Samples were carefully crumbled with a mortar and examined with a binocular loupe to search for *C. halimifolius* seeds. Only three groups of five pellets each were analysed in the deer faecal samples, and an equivalent sample mass in the wild boar, cattle and horse faeces. Found seeds were stored in paper envelopes (5.5 × 7 cm) in darkness at ambient temperature. Where seeds were found (*n* = 13), the whole remaining faecal sample was analysed, noting the final number of seeds. In most cases, the remaining faecal samples were not further examined. A reference collection of *C. halimifolius* mature and immature seeds and fruits was used to assist identification in the faecal samples (Fig. S2). Moreover, seeds were also carefully checked to assess whether they were damaged. Data on seed content of ungulate faeces were used to calculate the percentage of samples with seeds for each mammal species and study site.

### Seed germination experiment

No *C. halimifolius* seeds were found in faeces of ungulates other than red deer, except for one seed in wild boar faeces, which we did not consider in the seed germination experiment. Thus, we focused on red deer to assess their effects (positive or negative) on *C. halimifolius* seed germinability. We undertook germination experiments in chambers under controlled conditions using seeds retrieved from (*i*) red deer faeces and (*ii*) mature *C. halimifolius* fruits (i.e., ‘control’ seeds) collected within our study plots during summer 2023. All seeds were placed in Petri dishes (90 mm diameter) on a filter paper base, organized by sample, with distilled water (~11 mm^3^). We replicated the experiment in 2023 and 2024. In 2023, the experiment consisted of four dishes with control *C. halimifolius* seeds (12 seeds each) and three dishes with the experimental seeds from red deer faeces (4, 4 and 5 seeds, respectively). All experimental seeds were held in the germination chamber for 63 days. In 2024, the experiment consisted of 10 dishes with control *C. halimifolius* seeds (12 seeds each) and 15 dishes with experimental seeds from the nine deer faecal samples (up to 12 seeds per dish), using seeds found in the subset of faecal samples analysed in 2024. The experiment lasted 91 days. In both years, dishes in the chamber received 12 h light at 22°C, and they were checked for germination (i.e., seed coat break, radicle emergence) every 1–7 days, adding distilled water when necessary to wet the filter paper. Because of similarity in results of both experiments, and for simplicity, data from the two experiments were combined into a single dataset.

### Data analyses

To evaluate differences in frequency of visits to reproductive *C. halimifolius* by different ungulate species, we used camera traps and Pearson's *χ* ^2^ test. Camera traps indicated that red deer were, by far, the most dominant visitor. Therefore, we also assessed potential differences between study sites in red deer frequency of visits using the camera trap data. We fitted a generalized linear mixed model (GLMM) with Poisson error distribution using the R *lme4* package (R Core Team). Sampling day and camera id were included as random factors. We evaluated whether the effect of study site on number of red deer visits was significant using Likelihood ratio tests with *χ* ^2^ distribution using the R *lmtest* package (Lenth & Lenth 2018).

To identify potential differences between study sites in *C. halimifolius* seed content of faeces, the proportion of faecal samples with seeds in each study site was compared using Pearson *χ* ^2^ tests with the R *stats* package. Given that most faecal samples were not fully analysed, as only three groups of five pellets were examined per sample, we estimated the probability that the remaining unanalysed samples contained *C. halimifolius* seeds. We considered only red deer faecal samples (as they were the main species whose faeces contained seeds) analysed and seeds (*n* = 12). We then randomized the distribution of the found seeds across the groups of five pellets (i.e., between five and 14 groups of pellets per sample), with 20 iterations per faecal sample (overall, 240 iterations). For each iteration, we noted whether there were any seeds in the first three groups of pellets (similar to our procedure for incompletely analysed faecal samples). Then, we calculated conditional probability of finding seeds (given that the entire faecal sample contained seeds) within the first three group of pellets as the number of times that seeds were found in the first three groups of pellets/total number of iterations (*n* = 240).

To establish whether deer *C. halimifolius* seed consumption affected percentage germination, we compared cumulative percentages of germination of control and deer‐ingested seeds using Pearson *χ* ^2^ tests with the R *stats* package. In addition, to evaluate the effects of red deer ingestion on speed of seed germination, a Cox proportional hazards regression was conducted using the R *survival* package (R Core Team 2017). To separate the effects on germination speed from those on germination percentage, we only included germinated seeds in our analysis (e.g., Fedriani & Delibes 2009).

## RESULTS

### Frequency of ungulate visits

Overall, we photo‐recorded five ungulate species (wild red deer, fallow deer, wild boar) and (domestic horse, cattle) visiting our target *C. halimifolius* individuals (Table 2). There were significant differences in frequency of visits by different ungulate species (*χ* ^2^ = 1152.1, df = 5, *P* < 0.0001). Red deer were the most frequent visitor, both in Matasgordas (98.5% ungulate visits, *n* = 194; 98.6% ungulate individuals, *n* = 223) and in Reserva (68.3% ungulate visits, *n* = 139; 62.7% ungulate individuals, *n* = 169; Table 2, Table S2). There were significant differences between study sites in red deer frequency (*χ* ^2^ = 58.4, df = 1, *P* < 0.0001). This was higher in Matasgordas (1.06 red deer visits number of camera^−1^ day^−1^) than in Reserva (0.53 red deer visits number of camera^−1^ day^−1^). Of all red deer visits, 45.5% were recorded as non‐use and 54.5% as use of *C. halimifolius*. Wild boar was the second most frequent visitor, especially in Reserva where it represented 14.4% ungulate visits (Table 2); however, it was never recorded consuming *C. halimifolius*. Other ungulates (fallow deer, horse, cattle) were seldom recorded in our study plots (Table 2) and were never recorded using *C. halimifolius*, neither in Matasgordas nor in Reserva.

**Table 2:** Number and percentages of ungulate species visits recorded in camera traps in both study sites within the Doñana National Park (SW Spain).

species	matasgordas		reserva	
	number of visits	% total ungulates	number of visits	% total ungulates
Red deer	192	98.5	95	67.9
Fallow deer	1	0.5	3	2.1
Horse	0	0	20	14.3
Cattle	0	0	11	7.9
Wild boar	1	0.5	10	7.1

### Occurrence of *C. halimifolius* seeds in ungulate faeces

Overall, there were 114 seeds in 12 red deer faecal samples (4.5% faecal samples with seeds, *n* = 264). Only one seed was found in wild boar faeces (16.6%, *n* = 6). All seeds were mature, with a slightly smooth surface, but without structural damage. No *C. halimifolius* seed was found in horse and cattle faecal samples. Overall, there were nine and three red deer faeces samples with seeds from Matasgordas (3.1%, *n* = 139) and Reserva (1.1%, *n* = 125), respectively. There were no significant differences between sites in proportion of samples with seeds (*χ* ^2^ = 1.67, df = 1, *P* = 0.197).

According to our randomization approach using only fully analysed red deer faeces with seeds, 78% (187 times out of 240) of seeds were in the first three groups of five pellets. This indicates that analysing three groups of five pellets is a reasonable approach that balances lab effort and chance of finding seeds within faecal samples. However, in the remaining 22% (53 times out of 240) of instances where faecal samples contained seeds, these seeds did not occur in the first three groups of five pellets. Thus, the overall frequency of occurrence of *C. halimifolius* seeds in red deer faeces could be slightly higher than our estimated 4.5% (*n* = 264).

### Seed germination experiment

Overall, 87 seeds germinated (30.9%; *n* = 283) in sowing experiments in chambers, of which 68 were control seeds (40%; *n* = 168) and 19 were deer‐ingested seeds (17%; *n* = 114). There were strong significant differences in percentage germination between control and deer‐ingested seeds (χ^2^ = 16.9, df = 1, *P* < 0.0001), with mean accumulated percentage germination of control seeds being 1.9 times larger than that of deer‐ingested seeds. In 2023, one (8%, *n* = 13) and 45 (94%, *n* = 48) deer‐ingested and control seeds germinated, respectively. In 2024, 18 (18%, *n* = 101) and 23 (19%, *n* = 120) deer‐ingested and control seeds germinated, respectively.

**Fig. 2:**
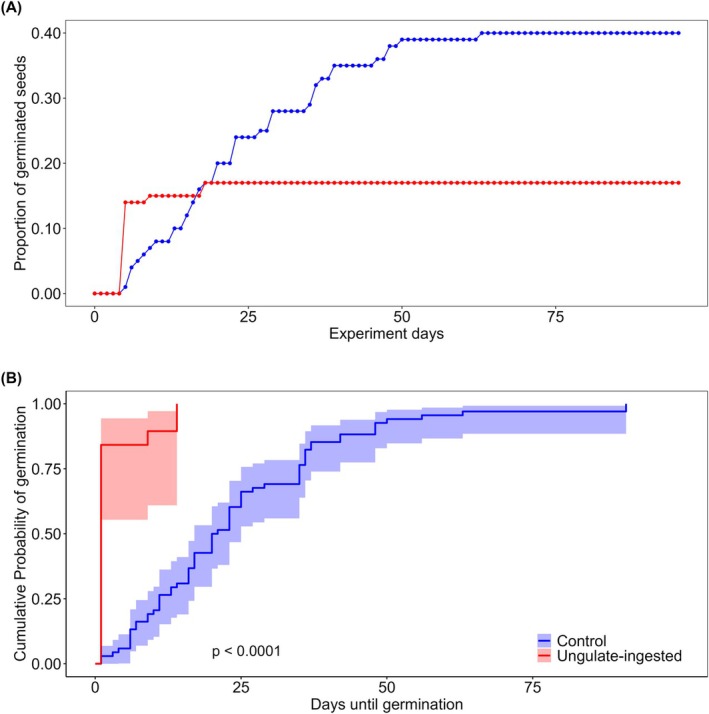
(A) Temporal trend of control and ungulate‐ingested *C. halimifolius* seeds. Both control (*n* = 168) and ungulate‐ingested (*n* = 115) groups consist of the cumulative sum of germinated seeds in 2023 and 2024. (B) Temporal trend of cumulative probability of germination of both control (*n* = 68) and deer‐ingested (*n* = 19) germinated seeds.

Control seeds started to germinate on day 5 of experiments, reaching a peak on day 63. Deer‐ingested seeds started to germinate on day 5 of experiments, reaching a maximum peak on day 18 (Fig. 2). Cox regression analyses indicated strong and significant differences between control and deer‐ingested seeds in germination speed, germination speed of deer‐ingested seeds being up to 12 times faster according to the hazard ratio (HR = 12.09, IC 95% = 6.24–23.45, *P* < 0.001).

## DISCUSSION

This is the first time in which the extent to which ungulates, red deer, have been evaluated as seed predators or seed dispersers of *C. halimifolius*. Our approach allowed us to identify whether plant–deer interactions were within rather than at the extremes of the mutualism–antagonism continuum (Perea *et al*. 2013; Bronstein 2015). Specifically, we propose that this interaction has a stronger antagonistic component than mutualistic component (i.e., long‐distance seed dispersal), which could be crucial for *C. halimifolius* population dynamics.

### *Cistus halimifolius* herbivory cost

Camera trapping confirmed the high frequency of ungulate interactions with *C. halimifolius*, with red deer by far the most frequent. The much fewer visits by other ungulate species to target *C. halimifolius* individuals may be related to the preference of these animals for other food sources, such as grass, which is more widespread in other areas of the territory (pers obs). Previous studies have shown that deer are intense browsers in Mediterranean landscapes (Bugalho & Milne 2003; Perea *et al*. 2014; Lecomte *et al*. 2016), so that in areas where their populations are high, as in Doñana National Park, scrubland vegetation is particularly affected. Evergreen shrub species, such as *C. halimifolius*, are an essential nutritional supplement for ungulates when pasture quality decreases in the dry season, providing easily digested food (Bugalho *et al*. 2001). The estimates from monitoring camera traps are consistent with data from the Natural Processes Monitoring Group of Doñana, showing that the deer abundance is higher in Matasgordas (Mean Index of Kilometric Abundance (IKA) of 8.52 ± 1.56) than in Reserva (IKA of 3.71 ± 0.38). These differences, together with the higher (1.7 times; unpubl. data) density of *C. halimifolius* in Matasgordas than in Reserva could explain these data, although presence of seeds within faecal samples was always low, it was slightly higher in Matasgordas faecal samples.

Faecal analysis revealed a very low percentage of *C. halimifolius* seeds from the examined samples. This could suggest that seed consumption by ungulates is infrequent. However, previous studies have shown that ungulates, especially deer, consume Cistaceae species at high rates (e.g., *Cistus ladanifer*; Malo & Suarez 1998; Bugalho *et al*. 2001; Bugalho & Milne 2003; Mussa *et al*. 2003; Lecomte *et al*. 2016; Agostini *et al*. 2020; García *et al*. 2023). Our camera trap monitoring revealed that red deer herbivory on *C. halimifolius* is intense. Indeed, in more than half of the red deer visits (54.5%) they seemed to be feeding on this plant. Based on personal observations, most *C. halimifolius* individuals simultaneously have flowers, immature fruits, and mature fruits. Unpublished data from 2023 to 2024 showed that red deer consume *C. halimifolius* fruits and seeds mostly at an immature stage, when seeds are softer and more vulnerable (Harada 1997; Ishikawa 2011). Thus, the asynchrony in the presence of the different reproductive structures of this plant likely explains the low presence of seeds in deer faeces, as consumption was mainly at the immature seed stage. Essentially, many ingested immature seeds were likely digested and destroyed during passage through the ungulate digestive tract and, consequently, not detected during our faecal analysis (e.g., Ishikawa 2011; Frost *et al*. 2013; Lecomte *et al*. 2017).

In general, small seeds, which are usually produced in higher quantities, as in *C. halimifolius*, are more efficient in escaping animal digestion (Traveset 1998; Pakeman *et al*. 2002; Mancilla‐Leytón *et al*. 2015). However, small seed size increases the mass‐to‐surface ratio and persistence in the digestive tract. This, together with a rough ridged seed coat, make *C. halimifolius* seeds more susceptible to gastric and intestinal fluids, enhancing their abrasion especially for immature seeds (Traveset & Verdú 2002; Mancilla‐Leytón *et al*. 2011; Milotić & Hoffmann 2016; Soltani *et al*. 2018). This may explain why, despite belonging to a family with hard and resistant seeds, seeds ingested by ungulates are not always able to germinate even under optimal conditions (Thanos *et al*. 1992; Ramos *et al*. 2006).

The results of germination experiments indicate that red deer reduced the germination ability of ingested mature *C. halimifolius* seeds, possibly for two different reasons. First, if mature seeds are retained in the deer digestive tract for a long time, embryos could be seriously damaged, losing ability to germinate (Gardener *et al*. 1993). Second, *C. halimifolius* seed structure and composition might be less resistant to digestion than other Cistaceae seeds, being more susceptible to mechanical, chemical or bacterial damage (Del‐Claro & Torezan‐Silingardi 2021), although this needs further investigation (Thanos *et al*. 1992). To comprehensively evaluate the extent to which immature and mature seeds are affected by the digestive tract, further germination experiments should be carried out with controlled animals directly fed with both immature or fully mature seeds (e.g., Mancilla‐Leytón *et al*. 2011, 2015).

### *Cistus halimifolius* benefits from herbivory

Several studies of Cistaceae seed dispersal (Mancilla‐Leytón *et al*. 2011) found that seeds resist passage through the digestive system of goats more than seeds of fleshy fruits. Furthermore, passage through the goat gut sometimes enhanced seed germination, softening the seed coat (Mancilla‐Leytón *et al*. 2015). This could explain results in our germination experiment, in which deer‐ingested seeds that managed to germinate do so 12 times faster, increasing seed survival by escaping from pathogens and predators or reducing competition with other species. Although prolonged sample storage could lessen germination capacity of our the deer‐ingested seeds, this does not seem a major issue, as the germination capacity of deer‐ingested seeds was higher in the 2024 experiment after prolonged sample storage.

Therefore, the *C. halimifolius*–ungulate interaction also benefits plant population dynamics. Red deer play an important role as effective seed disperser because of their high population densities in Doñana (9.32 individuals km^−2^) and many other Mediterranean ecosystems (up to 65 individuals km^−2^; Acevedo *et al*. 2008). Also, because of the large amount of faeces excreted daily (from 10.61 to 25.3 defecations per day; Horino & Nomiya 2008, Košnář & Rajnyšová 2013), even a relatively low percentage seed occurrence in faeces can result in huge numbers of dispersed seeds.

Barochory is the most frequent dispersal mechanism in Cistaceae (e.g., Bastida & Talavera 2002). However, a critical disadvantage of this type of dispersal is that it occurs over very short distances, so seeds fall and germinate in the immediate vicinity of the mother plant, which does not favour gene flow and dispersal to vacant habitats (Cheplick 1992; Thanos *et al*. 1992; Herrera 2002). This explains how the plant would benefit from a long‐distance seed dispersal mechanism. Red deer act as a potential long distance disperser, as they have large home ranges, from 200 to almost 1000 ha, and a minimum distance travelled per day from 3.0 to 4.2 km in the Iberian Peninsula (Carranza *et al*. 1991). Also, red deer intensively use (and defecate) in both open and closed habitats (Carranza *et al*. 1991). Therefore, the interaction of *C. halimifolius* with red deer can provide a fundamental benefit: long‐distance seed dispersal. This enables the plant to colonize vacant suitable habitats, and connect with other populations. Thus, maintaining gene flow, favouring long‐term population persistence (Levine & Murrell 2003; Nathan *et al*. 2008; Fedriani *et al*. 2012).

The known red deer browsing intensity (Perea *et al*. 2014) together with results from this study allow us to confirm, for the first time, the potential of red deer as long‐distance seed dispersers of *C. halimifolius* via endozoochory. This seed dispersal process relies on the successful completion of several stages: availability of diaspores, gut retention and germination, and establishment as seedlings (Wang & Smith 2002; Baltzinger *et al*. 2019). Therefore, further studies on these stages are necessary to fully assess the deer capacity for long‐distance dispersal. Accordingly, this investigation provides a global perspective on the dispersal of plants with dehiscent fruits and small and resistant seeds, such as *C. halimifolius* (Malo & Suarez 1998). Consequently, it broadens the range of dispersal mechanisms of plants adapted to environments similar to that in the Doñana National Park.

## CONCLUDING REMARKS

In this study we revealed that the red deer are simultaneously the main seed predator and long‐distance seed disperser of *C. halimifolius* in the Doñana area, reporting, for the first time, costs and benefits of this interaction which likely change in space and time. The consumption of fruits and seeds when still immature reduce plant dispersal and reproductive success. However, although less frequent, consumption of ripe fruits and mature seeds promotes long‐distance dispersal through endozoochory, allowing these seeds to reach distant habitats and connecting otherwise isolated shrub populations. Hence, the phenological stage of individual plants when they interact with red deer is decisive for seed dispersal success, and therefore, for the position of these plant–animal interactions within the mutualism–antagonism continuum. The sign and magnitude of this interaction is also likely affected by deer density (e.g., Lecomte *et al*. 2016; Jácome‐Flores *et al*. 2019; Muñoz‐Gallego *et al*. 2023) and spatio‐temporal variation. Thus, future studies should evaluate potential context‐dependent changes in the costs and benefits of this plant–ungulate interaction.

## AUTHOR CONTRIBUTIONS

RF‐F conducted fieldwork, processed samples, performed data analysis, and wrote the original manuscript. PJG participated in fieldwork and sample collection, and provided revisions to the manuscript. EV contributed to the revision of the manuscript. JMF reviewed the manuscript and secured funding for the project.

## Supporting information


**Fig. S1.** Transects carried out for the collection of faecal samples. (A) First transect and (B) total of transects made in Matasgordas. (C) First transect and (D) total of transects made in Reserva.

**Fig. S2.** Identification of seeds under the binocular loupe. (A) *C. halimifolius* seed found in a deer excrement. (B) Comparison of seeds found in excrements with a reference seed (red arrow). (C) Reference seeds extracted from *C. halimifolius* fruits.

**Table S1.** Total distance covered (km) and time spent (hours and minutes) per transect (Sampling day) in each sampling site.
**Table S2.** Summary of the number of faeces collected of each species per sampling day in both sampling sites.

## Data Availability

Data are available from the Figshare Repository: https://doi.org/10.6084/m9.figshare.29652203.
