# Supplementary figures and images for: The dual role of red deer in yellow rockrose seed dispersal and predation in Mediterranean Spain
Source: Plant Biol (Stuttg). 2025 Oct 3;27(7):1468–77. doi: 10.1111/plb.70102 (PMC12631517; doi:10.1111/plb.70102)

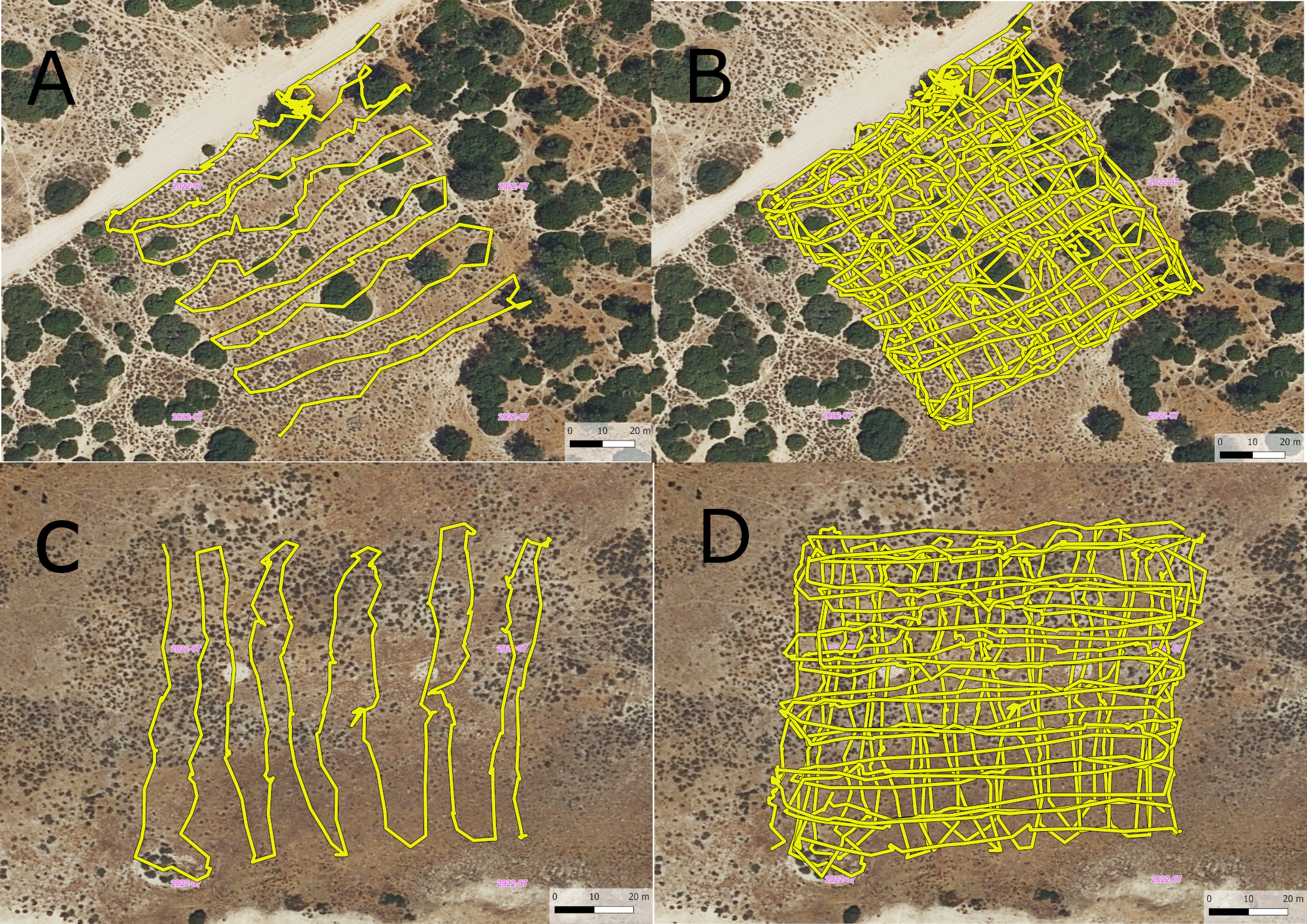

Supplement: Supplementary file 1 — Fig. S1. Transects carried out for the collection of faecal samples. (A) First transect and (B) total of transects made in Matasgordas. (C) First transect and (D) total of transects made in Reserva. [file PLB-27-1468-s003.png]

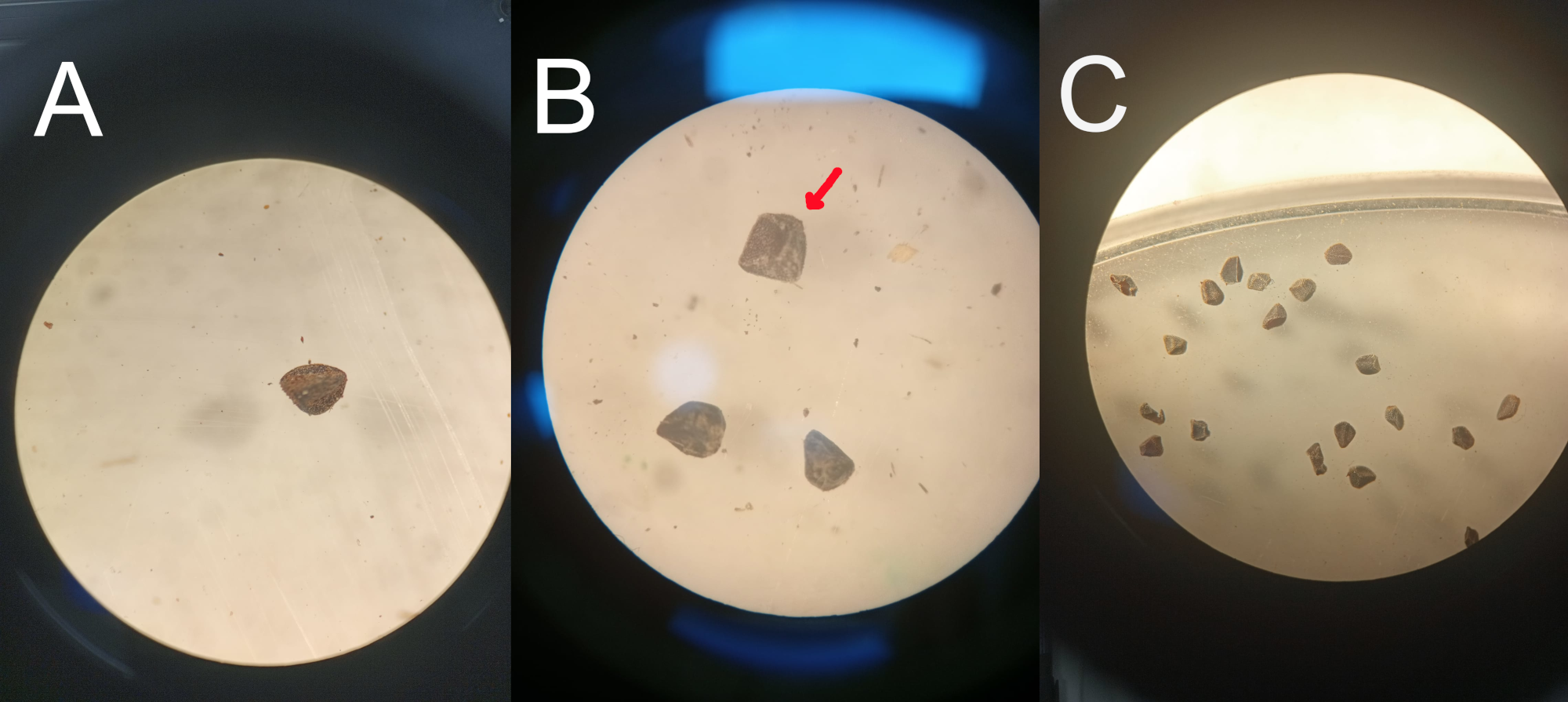

Supplement: Supplementary file 2 — Fig. S2. Identification of seeds under the binocular loupe. (A) C. halimifolius seed found in a deer excrement. (B) Comparison of seeds found in excrements with a reference seed (red arrow). (C) Reference seeds extracted from C. halimifolius fruits. [file PLB-27-1468-s001.png]
