# Supplementary material for: The dual role of red deer in yellow rockrose seed dispersal and predation in Mediterranean Spain
Source: Plant Biol (Stuttg). 2025 Oct 3;27(7):1468–77. doi: 10.1111/plb.70102 (PMC12631517; doi:10.1111/plb.70102)
Supplement: Supplementary file 3 — Table S1. Total distance covered (km) and time spent (hours and minutes) per transect (Sampling day) in each sampling site. Table S2. Summary of the number of faeces collected of each species per sampling day in both sampling sites. [file PLB-27-1468-s002.docx]

**Table S1**. Total distance covered (km) and time spent (hours and minutes) per transect (Sampling day) in each sampling site.

| Sampling Site | Sampling Day | Distance (km) | Time |
| --- | --- | --- | --- |
| Matasgordas | 1 | 1.48 | 2:26 |
|  | 2 | 1.29 | 2:06 |
|  | 3 | 1.17 | 2:06 |
|  | 4 | 1.11 | 2:20 |
|  | 5 | 1.03 | 2:12 |
|  | 6 | 1.11 | 2:06 |
| Reserva | 1 | 1.30 | 2:27 |
|  | 2 | 1.31 | 1:14 |
|  | 3 | 0.99 | 1:21 |
|  | 4 | 0.90 | 1:22 |
|  | 5 | 0.88 | 1:17 |
|  | 6 | 0.95 | 1:28 |

**Table S2.** Summary of the number of faeces collected of each ungulate species per sampling day in both sampling sites.

|  | | *Horse* | *Cattle* | *Deer* | *Wild boar* |
| --- | --- | --- | --- | --- | --- |
| Matasgordas | 1 | - | - | 74 | 1 |
|  | 2 | - | - | 55 | 0 |
|  | 3 | - | - | 60 | 5 |
|  | 4 | - | - | 83 | 0 |
|  | 5 | - | - | 65 | 0 |
|  | 6 | - | - | 69 | 1 |
| Reserva | 1 | 24 | 8 | 25 | 0 |
|  | 2 | 6 | 0 | 19 | 0 |
|  | 3 | 5 | 0 | 26 | 0 |
|  | 4 | 0 | 0 | 37 | 0 |
|  | 5 | 0 | 0 | 34 | 0 |
|  | 6 | 0 | 0 | 44 | 0 |
